# Developing Actinobacterial Endophytes as Biocontrol Products for *Fusarium pseudograminearum* in Wheat

**DOI:** 10.3389/fbioe.2021.691770

**Published:** 2021-06-29

**Authors:** Cathryn A. O’Sullivan, Margaret M. Roper, Cindy A. Myers, Louise F. Thatcher

**Affiliations:** Commonwealth Scientific and Industrial Research Organisation (CSIRO), Agriculture and Food, Floreat, WA, Australia

**Keywords:** biological control, crown rot, Actinobacteria, antifungal, cereal pathogen

## Abstract

Crown rot of wheat, caused by *Fusarium pseudograminearum*, results in millions of dollars of yield losses globally each year. Management strategies to control crown rot are limited and there are concerns about development of fungicide resistance so novel treatment strategies are desirable. A collection of endophytic Actinobacteria was screened for their ability to suppress the growth of *F. pseudograminearum* and the development of crown rot symptoms in wheat with the aim of identifying candidates that can be developed into biocontrol products. The ability of the Actinobacteria isolates to suppress the growth of three different *F. pseudograminearum* strains *in vitro* was assessed using agar-plate competition assays. Soil-free seedling assays were used to screen for suppression of development of early disease symptoms in the susceptible wheat (*Triticum aestivum)* cv. Tamaroi. Four of the isolates were tested in a glasshouse pot experiment to assess their ability to decrease disease symptoms and prevent yield losses in wheat cv. Tamaroi grown to maturity in an unsterilized soil. The screening of 53 isolates identified two *Streptomyces* isolates, MH71 and MH243, with very strong antifungal activity against *F. pseudograminearum* strains in agar-plate competition and seedling assays. In the glasshouse pot trial, plants treated with seed coatings of either MH71 or MH243 had > 24% lower disease severity than control plants infected with *F. pseudograminearum*. These two cultures show potential for development as biocontrol products because they are easy to culture, grow on relatively inexpensive media, produce highly durable spores and can be delivered to plants as a seed coat.

## Introduction

Fusarium crown rot is a fungal disease of cereals which occurs when the roots and crown of susceptible cereals become infected with one of several *Fusarium* spp., most commonly *Fusarium pseudograminearum* (Backhouse et al., 2004). Crown rot reduces both the yield and quality of cereals, with recent estimates showing that it costs growers ∼$80M for wheat and ∼$18M for barley annually in Australia alone (Murray and Brennan, 2009a,b). In winter wheat crops in the Pacific North West of United States, yield losses of 35% have been reported in commercial fields, while experimental plots experienced losses of up to 61% (Smiley et al., 2005).

The host range of Fusarium crown rot pathogens is wide and includes most sub-tropical and temperate cereals and grasses (Burgess et al., 2001). The highest economic losses tend to occur in wheat, barley and triticale (Burgess et al., 2001). Infection occurs at the crown (base of the stem) and decreases the ability of plants to transport water and nutrients from roots to shoots. Brown colored lesions with irregular margins (varying from honey/yellow through to deeper brown) on the lower stems and browning of the sub-crown internodes are diagnostic symptoms (Kazan and Gardiner, 2018). In many, but not all, cases plants develop white-heads (heads with little or no grain) because water, photosynthate and nutrients are prevented from being translocated (Burgess et al., 2001).

Yield losses vary between species and are strongly influenced by environmental factors, tending to be worse in growing seasons that begin wet and warm, promoting infection of young plants, and then become dry when the crop is setting seed, leading to water stress of plants that have impaired ability to take up water (Burgess et al., 2001). Crown rot instances tend to be worsened by successive cereal rotations, particularly in durum wheat cultivars (Lamprecht et al., 2006). Soil conservation practices that involve stubble retention also tend to be associated with increased crown rot risk because the pathogens survive over the summer fallow in stubble (Burgess et al., 1993).

Management strategies to control crown rot are somewhat limited. Fungicide seed dressings are available, but they are often inactivated over time by environmental conditions, soil physical properties or degradation by soil micro-organisms and so protection is usually best in the early stages of the plant growth cycle (Balmas et al., 2006). Plant breeding has produced some more resistant varieties but none of these show robust and durable disease resistance in the field; though genetic sources of resistance have been identified and resistance loci mapped (Liu and Ogbonnaya, 2015). An additional problem for crown rot control is that *F. pseudograminearum* and *F. culmorum* survive and continue to grow in the stubble left after harvest regardless of the level of resistance/tolerance of the wheat variety in the living phase of the crop, increasing the risk of infection in the following crop (Burgess et al., 2001; Simpfendorfer et al., 2003). These factors, combined with concerns about rising pathogen resistance to fungicides, consumer concerns about chemical loads in agriculture and off-target impacts, have led to increasing interest in novel approaches for the prevention or treatment of crown rot disease (Shimizu, 2011).

Biocontrol of plant fungal pathogens, using microbial inoculants, may form a highly useful component of integrated pathogen management programs. Biological products can provide growers with a treatment option that avoids issues with chemical residues on products (grains) and help to decrease the risk of pathogen resistance by providing a different mode(s) of action (Parnell et al., 2016). Several biological control products have been successfully commercialized to treat other crop diseases including products based on *Streptomyces lydicus* strain WYEC108 (registered as Actinovate by Novazymes BioAg Ltd. for treatment of powdery mildew, *Rhizoctonia* and *Phytopthora* root rots, and *Fusarium* wilt in a range of vegetables and flowers) and *Streptomyces griseoviridis* strain K61 Mycostop^®^ (registered as Mycostop by Verdera for the control of *Fusarium*, *Phytophthora*, *Alternaria* and *Pythium*, and for suppression of *Rhizoctonia* sp. and *Botrytis* sp. (Fravel, 2005; Shimizu, 2011; Junaid et al., 2013)). Studies from various locations around the world have noted the role of micro-organisms (bacteria and fungi) that produce secondary metabolites in the formation of disease suppressive soils (Weller et al., 2002). Such work suggests that there remains considerable unexplored potential within soil bacteria populations for further novel biocontrol products (Luo et al., 2014).

To be successful a biocontrol agent for soil-borne diseases must have a strong inhibitory effect on the target pathogen and be able to outcompete the native microbial community to persist in the rhizosphere. Actinobacteria have been recognized for many years for their ability to produce secondary metabolites which fight human and animal pathogens, forming the basis of a $30 billion industry that includes antibiotics, coccidiostats, animal growth promoters, anthelmintic and biopesticides (Demain, 2000). An added advantage of Actinobacteria is that some species are known to live as endophytes, taking up residence in plant roots, meaning that they are more likely to persist and exert an influence in the area where the pathogens are attacking the plant. For this reason there is an ongoing interest in screening microbial communities for novel species and strains that can suppress plant pathogens and reduce crop losses due to disease (Franco et al., 2007).

The aim of this investigation was to curate and screen a culture collection of Actinobacteria isolates for their ability to suppress the growth and disease expression of *F. pseudograminearum*. It was hypothesized that:

1. The culture collection would contain isolates capable of inhibiting the growth of *F. pseudograminearum in vitro*.
2. These isolates would provide protection of susceptible wheat plants from infection by *F. pseudograminearum* when applied as a seed coat.

A series of *in vitro*, seedling and glasshouse experiments were used to test these hypotheses in order to identify potential candidates for development as biocontrol agents for Fusarium crown rot.

## Materials and Methods

### Actinobacteria Cultures

Endophytic, plant-growth promoting Actinobacteria were isolated from the roots of field grown wheat plants in the wheat belt of Western Australia (WA). Wheat plants from areas of crop fields that showed consistent high-performance in terms of plant health and yield were selected for isolation. Cultures were isolated from sterilized root tissues of the sampled, healthy plants to target endophytic microorganisms.

Plant roots were washed with tap water to remove soil and the plant shoots were removed using scissors. After draining on paper towels, roots were surface sterilized by washing for 60 s in 99% ethanol, followed by 6 min in 3% NaOCl, and another 30 s in 99% ethanol. The roots were then rinsed in sterile distilled water, dipped in ethanol, flamed and then immediately extinguished by placing in a sterile, Pyrex petri dish with a lid on. The roots were then sectioned aseptically (with flamed scalpel and tweezers) into ∼1 cm long fragments and placed on Mineral Salts 2 solid media containing NaNO_3_ 2 g/L, K_2_HPO_4_ 1 g/L, KH_2_PO_4_ 0.5 g/L, KCl 0.1 g/L, MgSO_4_.7H_2_O 0.5 g/L, CaCl_2_ 0.01 g/L, FeSO_4_.7H_2_O 0.01 g/L, Na EDTA 0.0015 g/L, Yeast Extract 3 g/L (Akit et al., 1981), glucose 20 g/L, agar 15 g/L, and Benlate 50 mg/L (to suppress fungal growth).

The plates were incubated at 30°C in the dark for 4 weeks. Plates were checked weekly for the appearance of colonies. Actinobacteria are known to be slow growing so it was expected that the colonies would appear after several weeks of growth. Actinobacteria colonies formed at the cut ends of the plated root fragments. The colonies were selected based on expected morphology, with the most common Actinobacteria colonies appearing as dull, opaque, raised, hard, powdery colonies that have a mat like finish and are hard to pull off plates. Actinobacteria may vary in the production of colored compounds including blue, purple, black, red, orange, and yellow. Other morphological characteristics used for selection included flat, circular, waxy, blurry, and/or pimple like colonies.

Suspected Actinobacteria colonies were picked, by touching the center of a colony with a sterile wire needle, and plated onto isolation media (half strength potato dextrose agar (HPDA, 19.5 g/L Oxoid PDA dehydrated culture media plus 7.5 g/L Oxoid bacteriological agar)). Isolation plates were incubated at 30°C and growth was monitored to ensure only one colony morphology appeared. Further selection of isolates produced approximately 300 individual isolates of Actinobacteria.

A selection of the cultures, 53 in total, were considered suitable for screening for suppression of *F. pseudograminearum* in *in vitro* fungal suppression and *in planta* tests. These isolates were readily culturable and produced sufficient spores that could be introduced to plants via a seed coat, both critical criteria for development of a commercial biocontrol product (Parnell et al., 2016). All the Actinobacteria cultures were grown on half strength potato dextrose agar plates at 28°C. Spores were harvested from mature colonies and stored as a suspension in sterile distilled water at room temperature for use as inocula for all experiments.

*F. pseudograminearum* cultures were kindly provided by Dr. Kemal Kazan, CSIRO Agriculture and Food, and were originally isolated from crown rot infested wheat plants collected from southern Western Australia. Three highly virulent strains of *F. pseudograminearum* were tested, CS3427, CS5834, and CS5642 (Moolhuijzen et al., 2013; Sabburg et al., 2015; Powell et al., 2017). Spores of the *F. pseudograminearum* cultures were stored as a suspension in sterile distilled water at room temperature. *F. pseudograminearum* isolates were grown on HPDA plates at 28°C to provide inocula for the plate competition assays or in modified V8 vegetable juice broth (200 mL.L^–1^ V8 juice, 800 mL.L^–1^ sterile distilled water, 4 g.L^–1^ CaCO_3_; Alfonso et al., 1992) incubated at 28°C in a shaker incubator rotating at 70 rpm for use as inoculum for the seedling assays and pot trials.

### Identification of Actinobacterial Isolates by 16s rRNA Gene Sequencing

The 16s ribosomal DNA of a subset of the isolates were sequenced and compared to existing databases for identification. Each isolate was grown on a HPDA plate until vegetative mycelia formed. The mycelia were collected aseptically, and DNA was extracted using a MoBio PowerSoil DNA extraction kit, following the manufacturer’s protocols (MoBio, Qiagen, United States). The 16s rDNA was amplified using the Actinobacteria specific primers described by Monciardini et al. (2002) (243F 5′-GGATGAGCCCGCGGCCTA-3′; A3Rb 5′-CCAGCCCCACCTTCGAC-3′) using Bioline Immolase PCR Mastermix (Bioline, Australia). PCR conditions consisted of a hot start at 95°C for 4 min followed by 30 cycles of 94°C for 1 min, 48°C for 1 min, and 72°C for 2 min and a final step of 72°C for 10 min.

The PCR product was checked for quality by running an electrophoresis gel, and product of sufficient quality was sent to the Australian Genome Research Facility (AGRF) for sequencing using the same primer set described above. Sequences returned from AGRF were checked for quality and cropped using Chromas software (Technelysium, Australia) and then compared to the NCBI Basic Local Alignment Search Tool (BLAST) database to identify close relatives based on the 16s rRNA sequences (Altschul et al., 1990).

Sequences of representative near relatives, identified from the BLAST output were downloaded from NCBI GenBank (Benson et al., 2011). The sequences were aligned in the MEGAX software package (Kumar et al., 2018). A phylogenetic tree was created using the Maximum Likelihood method and Tamura-Nei model (Tamura and Nei, 1993). Initial tree(s) for the heuristic search were obtained automatically by applying Neighbor-Join and BioNJ algorithms to a matrix of pairwise distances estimated using the Tamura-Nei model, and then selecting the topology with superior log likelihood value. This analysis involved 26 nucleotide sequences. Codon positions included were 1^st^ + 2^nd^ + 3^rd^ + Non-coding. There were a total of 1,577 positions in the final dataset. Evolutionary analyses were conducted in MEGA X (Kumar et al., 2018).

### *In vitro* Fungal Suppression Tests

*In vitro* competition tests were conducted by inoculating one end of a HPDA plate with a test Actinobacteria isolate and other end with a plug of *F. pseudograminearum* as described by Coombs et al. (2004).

Actinobacteria were cultured onto the top 1/4 of HPDA plates from spores stored at room temperature in sterile distilled water by dipping a sterile inoculation loop into the spore suspension and spreading across the defined area of the plate. Plates were incubated at 28°C in the dark until a lawn of Actinobacteria was established over the top third of the plate (approximately 2 weeks). The opposite end of the plate was then inoculated with a small plug, approximately 1 cm diameter, cut from a HPDA plate with a healthy lawn of *F. pseudograminearum*. A plug of each *F. pseudograminearum* strain was also inoculated onto a clean HPDA plate as a control to assess uninhibited growth of the pathogen under test conditions. Triplicate plates were set up for each Actinobacteria-Fusarium combination and incubated at 28°C.

When the control plate was completely covered by fungal growth (approximately 1 further week of incubation) the experimental plates were examined. Plates were photographed and the distance (mm) between the growing front of the test Actinobacteria and the fungal pathogen, known as the “zone of suppression,” was measured as an indication of antifungal activity.

### Seedling-Scale, *in planta* Screening

The ability of the antifungal isolates to suppress crown rot disease *in planta* was initially investigated under soil-free conditions using a modified paper towel assay (Yang et al., 2010). Wheat cv. Tamaroi, a durum wheat known to be susceptible to crown rot infection, was used for testing (Chakraborty et al., 2010). Prior to seed coating, wheat seeds were surface sterilized by soaking in 3% sodium hypochlorite solution for 5 min, followed by thorough rinsing in sterile distilled water and drying in a laminar flow cabinet. Seeds were then coated with Actinobacteria spores by mixing the seeds and spores in a 0.3% xanthan gum solution under aseptic conditions. The coated seeds were dried in a laminar flow cabinet. Once dried, the coated seeds could be stored at room temperature. A sub-sample of each xanthan gum spore suspension was used to create a serial dilution series that was plated out to determine the viable cell count of each Actinobacteria treatment. The Actinobacteria treatments and viable spore counts in the seed coats are listed in Supplementary Table 1. Disease controls, i.e., uncoated seeds (no Actinobacteria) challenged with *F. pseudograminearum* CS5642, and healthy controls, i.e., uncoated seeds with no pathogen, were included for comparison with the biocontrol treatments.

For each Actinobacteria treatment, 25 coated seeds were allowed to imbibe sterile water in a Petri dish for 2–3 days prior to testing. Once root radicals were visible, five seeds were rolled in damp paper towels to form “cigars” with the seeds at the top of the paper towel roll. Five rolls were set up for each treatment giving a total of 25 replicate plants. The seeds within the paper rolls germinated standing upright in a beaker containing 50 mL of water. Water levels were checked daily and topped up with sterile water to maintain 50 mL.

**FIGURE 1:**
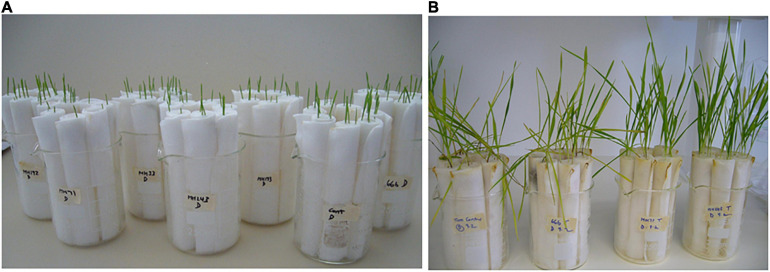
Seedling-scale assay: *F. pseudograminearum* infected seedlings of wheat cv. Tamaroi at the time of inoculation **(A)** and at the time of scoring disease severity **(B)**.

After germination (shoot emergence rate ∼90%, Figure 1A), the rolls of wheat seedlings were inoculated with 2 mL of a suspension of *F. pseudograminearum* CS5642 spores (10^6^.mL^–1^) applied to the roots. Inoculated seedlings were placed in a growth cabinet set at 10 h light, 14 h dark/24°C day, 15°C night.

The seedlings were grown for 2 weeks (Figure 1B) and then examined for evidence of infection. Shoot and root length, and the percentage of stem browning, as a measure of disease severity (Obanor et al., 2013), were recorded for each seedling.

### Glasshouse Pot Experiment

A pot experiment was undertaken under controlled environment conditions to assess the efficacy of the Actinobacteria seed coats in suppressing crown rot disease in wheat plants grown to maturity and preventing yield loss.

The experiment was conducted in an evaporatively-cooled glasshouse in Perth, (WA, 31.95^*o*^S, 115.79^*o*^E), with day/night temperatures of 32/10 (°C), and average max/min relative humidity of 90/30% (Supplementary Figure 1). The glasshouse had a natural photoperiod (minimum day length of approximately 10 h in June; maximum day length approximately 13.5 h in early November) and an average maximum photosynthetic photon flux density (PPFD) of 952 ± 21μmol m^–2^ s^–1^ measured at the plant level at 13:00 h. Temperature and humidity records for the duration of the experiment are supplied in Supplementary Material.

Based on the results from the *in vitro* assays and seedling disease suppression assays, four Actinobacterial isolates, MH71, MH243, MH192, and 66b, were selected for glasshouse scale testing. Sterilized wheat, cv. Tamaroi, seeds were coated with Actinobacteria spores in 0.3% xanthan gum as described above. Disease and healthy controls were setup as described for the seedling assay.

Two seeds of each treatment were planted into cylindrical PVC pots (25 cm diameter, 45 cm high) with 20 replicate pots for each treatment. The soil was an unsterilized, dark brown, sandy loam (a Tenosol as defined by *Isbell*; Isbell, 2016) from Gingin, Western Australia (31.34°S, 115.91°E) with a near neutral pH [6.77 ± 0.03 (CaCl_2_)], a low NPK content, low organic carbon and low clay/silt content (Duncan et al., 2018). Prior to sowing, the soil was saturated with water and allowed to drain for 24 h to give a water content approximating field capacity. Following sowing, all pots were watered 3 times per week with 150 mL water. Low doses of fertilizer (0.075 g/pot of Thrive Soluble All-Purpose Plant Food; Yates, Australia) were applied to all pots at 2, 4, 6, and 8 weeks after sowing.

When the plants reached the 2-leaf stage (approximately 2 weeks) each pot was inoculated by applying approx. 5 g of finely ground cracked corn infected with *F. pseudograminearum* CS5642 to the soil surface around the plant crown (Melloy et al., 2014).

At anthesis (~16 weeks after sowing) one plant was harvested from each pot, and shoot length, length of stem browning and disease severity (% stem browning) were recorded for each plant. At maturity (~25 weeks after sowing) the other plant was harvested from each pot and the same measurements were made. In addition, above-ground biomass and grain yield were recorded.

### Statistical Analysis

The size zones of suppression in the *in vitro* assays and the stem browning data from the seedling assays and pot trial were analyzed for statistical significance by comparing each treatment with the disease control using a one-way ANOVA (Genstat 16th edition 2016). Due to the small number of grain heads produced in the pot trial, grain samples were pooled during harvest, so it was not possible to test the statistical significance of these differences because of the lack of replication.

## Results

### Sequencing of 16s rDNA

**FIGURE 2:**
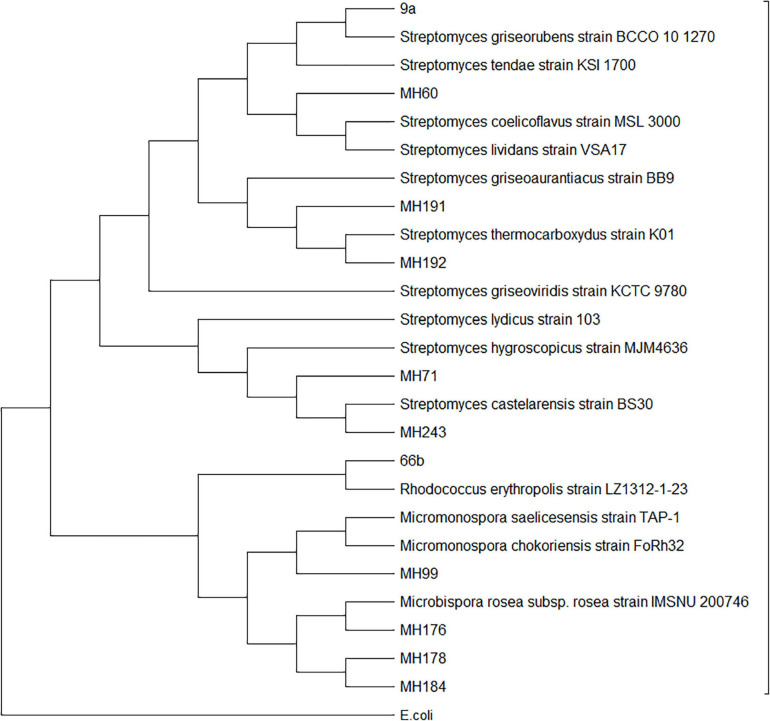
Molecular phylogenetic analysis of Actinobacteria isolates based on 16s rDNA sequences by Maximum Likelihood method.

Twelve of the 53 Actinobacteria isolates were selected for 16s ribosomal DNA sequencing to confirm their identity. Isolates with differing colony morphology (Supplementary Table 2 and Supplementary Figures 2–5) were selected in an effort to provide a diversity of genera. The BLAST results and subsequent phylogenetic analysis of the sequences grouped the isolates into expected genera considering their colony morphology on HPDA plates (Figure 2 and Supplementary Figures 2–5). Isolates 9a, MH60, MH191, MH192, MH71, and MH243 all clustered within the *Streptomyces* genus. MH176, MH178, and MH184 clustered with *Microbispora*. MH99 fell within *Micromonospora* and 66b fell within *Rhodococcus*. MH71 and MH243 proved to be closely related to each other and clustered with previously identified species known to produce antifungal metabolites, i.e., *Streptomyces hygroscopicus* and *Streptomyces lydicus* (Barka et al., 2016). Taking into account the closeness to Streptomyces castelarensis, this would define MH71 and MH243 as members of the Streptomyces hygroscopicus clade (Rong and Huang, 2012). MH191 and MH192 also were closely related to each other and clustered with species known to produce antifungal metabolites, i.e., *Streptomyces griseoviridis* (Kortemaa et al., 1994). The 16s rDNA sequences have been uploaded to the Genbank database and accessions are noted in Supplementary Table 2. The most promising isolates (MH71 and MH243) have been deposited to an Australian Culture Collection at the National Measurement Institute (Port Melbourne, Victoria, Australia).

### *In vitro* Fungal Suppression Tests

A total of 53 isolates were screened for antifungal activity against *F. pseudograminearum* CS5642 using the *in vitro* plate assay (Supplementary Table 3). A selection of the isolates was also tested against two other *F. pseudograminearum* strains, CS5834 and CS3427. The remaining 250 isolates in the original collection either grew too slowly or did not produce enough biomass/spores to allow for testing or for production of a seed coating (minimal growth after 4 weeks on a variety of different solid media at 26°C).

**FIGURE 3:**
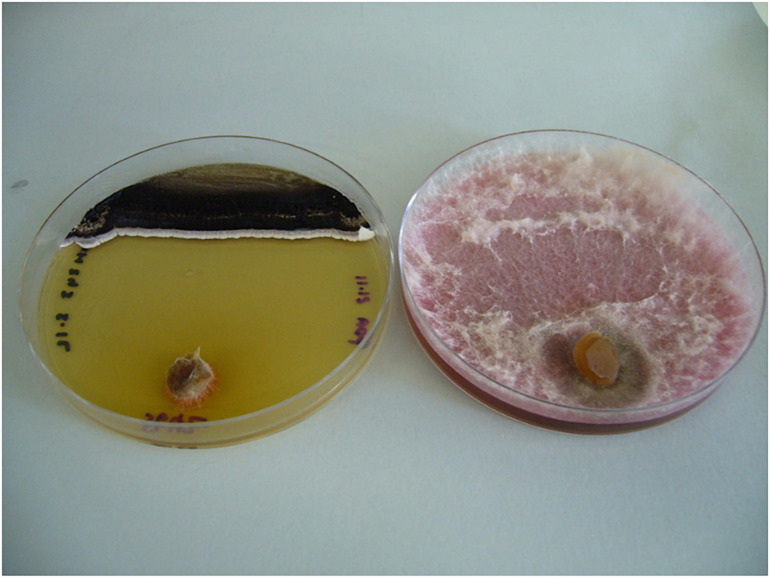
MH243 (black lawn at top of left hand plate) inhibited growth of *F. pseudograminearum* CS5642 (inoculated via plug at bottom of both plates) compared with control plate containing *F. pseudograminearum* CS5642 alone (on right).

**FIGURE 4:**
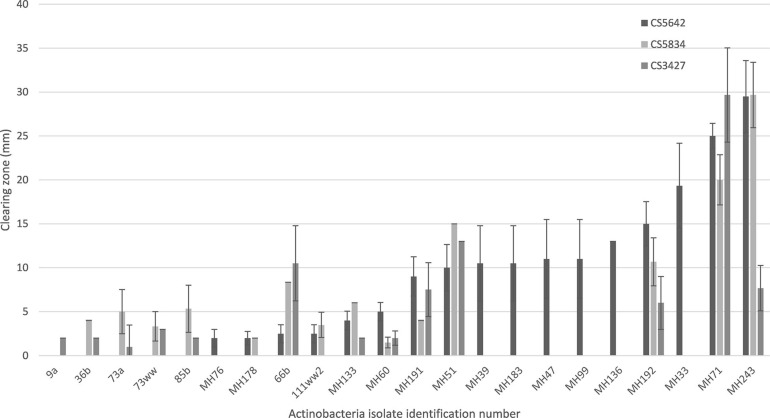
Selected results for the suppression of growth of three different strains of *F. pseudograminearum* by a range of Actinobacteria isolates measured as suppression zones (mm) on *in vitro* plate tests. Results for isolates with non-zero suppression zones against at least one *F. pseudograminearum* strain shown. Full data set is shown in Supplementary Table 3.

Of the isolates tested, MH71 and MH243, demonstrated very strong antifungal activity against all 3 strains of *F. pseudograminearum*, almost completely suppressing growth on HPDA plates (Figures 3, 4). A further 9 isolates, strongly suppressed the growth of at least one of the fungal strains (zone of suppression > 15 mm), while another 10 isolates were moderately suppressive against at least one *F. pseudograminearum* strain (with suppression zones of between 5 and 15 mm). The remainder either caused minimal suppression (<5 mm) or did not suppress the growth of *F. pseudograminearum* at all (Figure 4 and Supplementary Table 3).

### Seedling-Scale, *in planta* Screening

**FIGURE 5:**
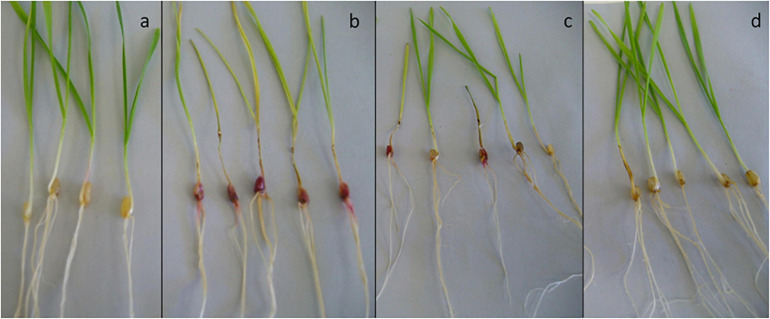
Example photos of seedling disease protection assays. Wheat cv. Tamaroi seeds were coated with xanthan gum containing spores of a treatment Actinobacteria (or xanthan gum alone for controls) prior to germination. Seedlings were challenged with a 2 mL of a broth containing mycelia of *F. pseudograminearum* CS5642 (at the 2-leaf stage of growth). Pink/red discoloration and stem browning indicate *F. pseudograminearum* infection. **(a)** healthy control; **(b)** disease control; **(c)** seeds treated with ineffective inoculant 66b; **(d)** seeds treated with highly beneficial MH71.

Based on the results of the *in vitro* plate assays, 20 isolates were selected for testing for their ability to protect wheat seedlings against *F. pseudograminearum* CS5642 infection. Disease control seedlings (those infected with *F. pseudograminearum* only) all developed severe crown rot symptoms, with stem browning as well as pink mycelia visible on the seeds (Figure 5b).

**FIGURE 6:**
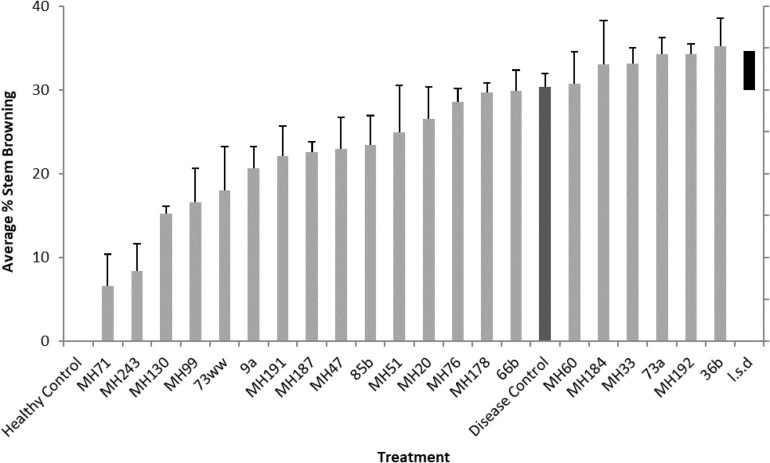
Crown rot symptoms (% stem browning) observed on seedlings grown from seeds coated with Actinobacteria isolates and inoculated with *F. pseudograminearum* CS5642. L.s.d denotes least significant difference (at *P* = 0.05) in expression of disease (% stem browning).

**FIGURE 7:**
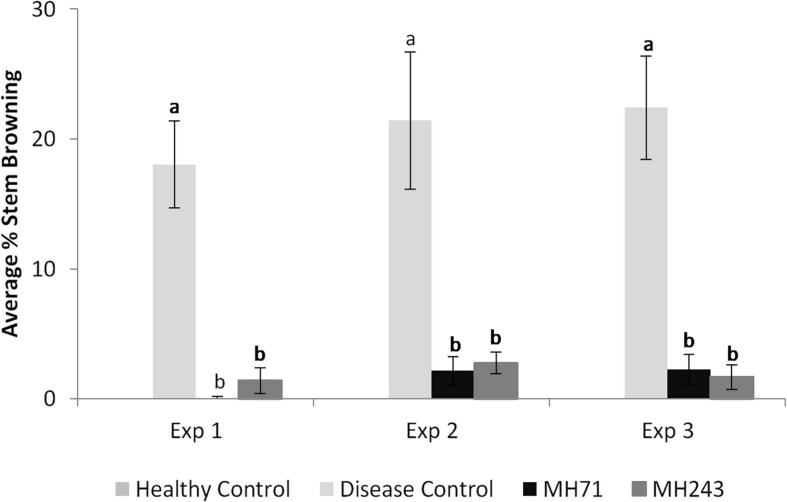
Crown rot symptoms (% stem browning) observed on seedlings grown from seeds coated with Actinobacteria (MH71 and MH243) and inoculated with *F. pseudograminearum* CS5642 in 3 separate seedling-scale experiments. Inoculation with MH71 or MH 243 decreased disease expression by between 75 and 100%. Different letters (a,b) denote significant difference at *P* < 0.05 from ANOVA analysis.

Nine of the Actinobacteria isolates significantly reduced disease severity in seedlings, measured as percent stem browning (Figures 5, 6). Isolates MH71, MH243, MH130, and MH99 gave the greatest protection from disease expression in seedlings (Figure 6). Repeat testing of the 2 most suppressive Actinobacteria isolates (MH71 and MH243) showed consistent results across 3 repeat experiments (Figure 7). In separate tests, MH71 and MH243 were introduced onto sterilized wheat seeds as a seed coating and then re-isolated from the roots of the emerged seedlings proving that the actinobacterial endophytes colonized the roots of the plant.

### Glasshouse Pot Experiment

Four isolates were chosen for inclusion in the glasshouse pot experiment. MH71 and MH243 were selected because they consistently produced evidence of high levels of suppression of *F. pseudograminearum* in the *in vitro* and *in planta* seedling assays. Isolates 66b and MH192 were chosen at random to represent less suppressive isolates.

**FIGURE 8:**
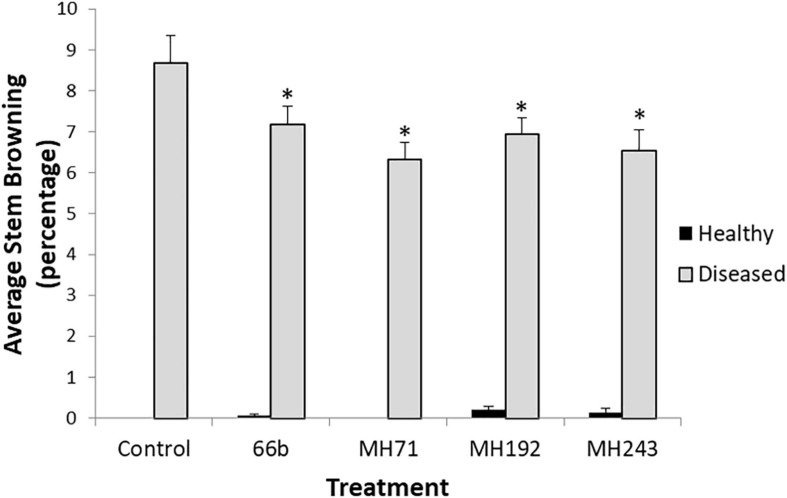
Disease severity (% stem browning) at maturity in the glasshouse experiment. ^∗^ Indicates significant difference (*P* < 0.05) between the disease control and disease infected plants treated with Actinobacteria seed coats. Healthy plants received the same Actinobacterial seed coat treatments (or plain xantham gum coating for controls) but were not challenged with *F. pseudograminearum* CS5642.

For all 4 isolates, plants treated with seed coats prior to planting had significantly lower stem browning at harvest than the disease control (*P* < 0.05) (Figure 8). Disease severity was relatively low compared to the seedling tests but application of the Actinobacterial treatments resulted in statistically significant reductions in stem browning (Figure 8). None of the plants developed whiteheads during the pot experiment, despite significant stem browning.

**FIGURE 9:**
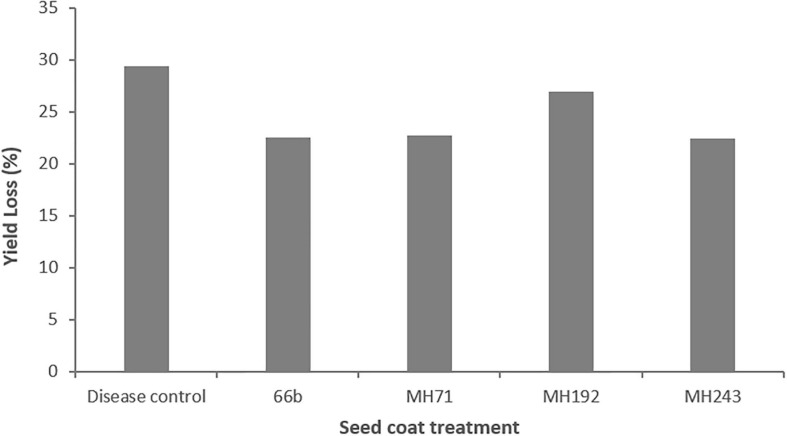
Grain yield losses in plants infected with *F. pseudograminearum* and treated with a range of Actinobacteria isolates as seed coats in a glasshouse pot experiment. Losses are expressed as a percentage compared to healthy control plants that were grown under the same conditions but not infected with *F. pseudograminearum*.

Due to low grain head production across the experiment, grain samples per treatment were pooled for analysis. This meant that statistical analysis could not be performed on this data due to lack of replication and it must, therefore. be considered as preliminary. Grain yields were impacted by crown rot infection (Figure 9). The disease control plants yielded 29% less grain than healthy control plants without *F. pseudograminearum* infection. Plants treated with Actinobacteria seed coats showed evidence of protection from yield loss, with plants treated with MH71, MH243 and 66b suffering reduced yield losses of 22.7, 22.5 and 22.5%, respectively (Figure 9). Plants treated with MH192 suffered yield losses of 27%, similar to the losses in the disease controls (Figure 9).

## Discussion

At present there are no wheat varieties with high levels of resistance to *F. pseudograminearum*, so growers rely on an integrated management approach combining tillage and stubble management, crop rotation and the use of partially resistant varieties (Chakraborty et al., 2010). Despite their efforts, growers continue to suffer significant losses due to crown rot (Smiley et al., 2005; Murray and Brennan, 2009a,b). There is also some concern that some *Fusarium* species are developing resistance to existing fungicide treatments (Lucas et al., 2015) and there are growing consumer concerns about the use of chemicals in food production (Shimizu, 2011). All these factors are driving interest in alternative control strategies.

The study reported here used a bio-guided, natural product screening approach to look for novel antifungal treatments. This approach involves untargeted screening of isolates for their ability to suppress or inhibit pathogens of interest. Lyddiard et al. (2016) argue that modern, genetic and target-focussed methods of antibiotic discovery have not been as productive as expected and that modernizing the simple, bio-guided assays that were used in early antibiotic discovery could be a more effective screening method for discovering novel compounds. The results of our screening study show that it is possible to find candidate biocontrol organisms that are highly effective at suppressing plant pathogens using simple, bio-guided assays. However, combining this approach with genomic tools will be critical if we are to fully understand the identity of the organisms and their modes of action, information that will be required for full optimization of the production of active compounds.

The 16s rDNA sequencing and phylogenetic analysis conducted on the isolates in this study revealed that several of the most suppressive bacteria belonged to the Streptomyces genus, and grouped with species that have been successfully developed as biocontrol products in the past. MH71 and MH243 clustered with *Streptomyces hygroscopicus*, which produces a range of antibiotics including for some strains rapamycin, nigericin and validamycin (Lee et al., 2016; Yoo et al., 2017) and *S. lydicus*, which has been commercialized for the control of plant fungal pathogens as Actinovate (Novazymes BioAg Ltd) (Chen et al., 2016). MH191 and MH192 clustered with *S. griseoviridus*, commercialized as a plant antifungal called Mycostop (Sabaratnam and Traquair, 2002), and *S. grisaurantiacus*, which produces the antibacterial compound diperamycin (Matsumoto et al., 1998). It is not surprising that the isolates tested in this study cluster with other Actinobacterial species that have been shown to produce bioactive secondary metabolites, but it should be noted that closely related organisms will not necessarily have similar abilities to produce secondary metabolites (Sottorff et al., 2019). Despite their phylogenetic relationships, it is possible that both these isolates and the compounds that they produce are novel.

While the 16s rDNA sequencing and phylogenetic analysis identified the genera of the isolates, it was not possible to provide definitive identification at the species level, a common problem for Actinobacteria (Labeda et al., 2012, 2017). Whole genome sequencing, using comparisons based on multiple loci, is needed to enable identification at the species level. Whole genome data could also be used for bottom-up screening for natural products based on gene clusters coding for putative antifungal compounds which could be expressed or synthesized (Luo et al., 2014).

Several of the Actinobacteria isolates described in this screening study strongly inhibited the growth of *F. pseudograminearum* on HPDA plates and suppressed the development of crown rot symptoms in wheat. Two isolates in particular, MH71 and MH243, showed potential for development as preventative treatments for crown rot caused by *F. pseudograminearum* in wheat. These two isolates almost completely inhibited the growth of three different strains of *F. pseudograminearum* on HPDA plates (Figures 3, 4). Wheat seedlings treated with either of these two isolates exhibited reduced severity of infection by *F. pseudograminearum* in the seedling assay (Figures 5–7). In the pot trial, wheat plants treated with either MH71 or MH243 had 25 and 24% less stem browning, respectively (Figure 8). While the lack of replication and statistical analysis means that the yield data must be considered as preliminary, the trends agreed with results of the disease severity assessment. Plants treated with either MH71 or MH243 suffered ∼16% less yield losses than the disease control plants (Figure 9). These decreased yield losses are in the same order of magnitude as those achieved in field trials using management interventions including sowing on the inter-row, residue management and ground engaging tools (Verrell et al., 2017). Combining these treatments with biocontrol seed coats could be of value as a disease management approach. However, this yield data must be treated with caution due to the lack of statistical analysis. The focus of this work was an analysis of the impact of biocontrol treatments on disease severity, but future work should include investigation of the impact of the biocontrol candidates on grain yield and quality both in pots and in the field over multiple generations and sites.

There are few available reports on the effect of biocontrol treatments on wheat infected with *F. pseudograminearum*. Stummer et al. (2020) assayed wheat seed inoculated with *Trichoderma gamsii* strains Tk7a and A5MH and strain *T. harzianum* Tr904, and recorded a 12–32% increase in wheat biomass at maturity relative to *F. pseudograminearum*-only controls but did not report on any grain yield benefit. Lounaci et al. (2016) reported a significant suppression of crown rot disease due to *F. culmorum* and increases in the root and shoot weight of wheat seedlings treated with the rhizobacterium *Paenibacillus polymyxa* but their experiments were conducted on young plants and they did not report changes in grain yield. Several papers report significant decreases in disease severity and yield loss due to *F. graminearum*, the causal agent of Fusarium head blight, through the application of antagonistic microbial treatments (Huang and Wong, 1998; Schisler et al., 2002) but these results are not directly comparable to plants infected with *F. pseudograminearum*.

Parnell et al. (2016) state that biocontrol products currently make up a relatively small proportion (3.5%) of the US$ 1.6 billion global pesticide market but sales of biopesticides are growing, increasing from US$0.5 billion in 2009 to US$1.09 billion in 2015 in North America and Western Europe alone. Growth of markets for biocontrol products are expected to be significantly stronger than synthetic pesticides in coming years (Glare et al., 2012).

Actinobacteria have been known as a source of antifungal and antibacterial secondary metabolites for many years (van Wezel and McDowall, 2011). The majority of commercialized compounds from Actinobacteria are used for protecting human health (Demain, 2000; Lancini and Demain, 2013) but there is growing demand for biological products to help in the control of recalcitrant crop diseases like Fusarium crown rot.

**FIGURE 10:**
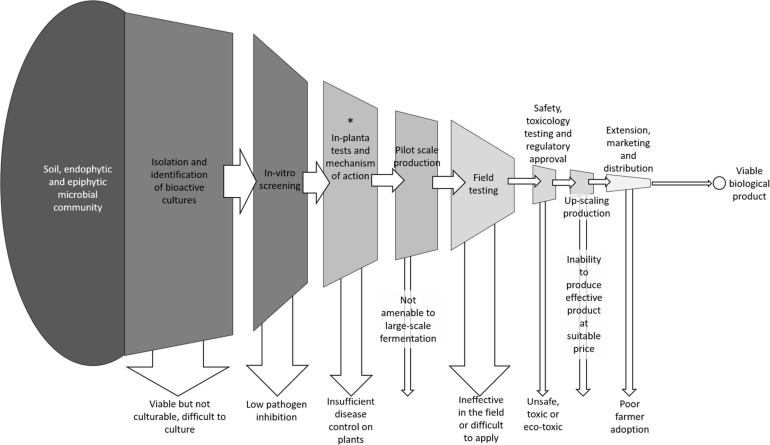
Conceptual diagram of the steps involved in bringing a new biological disease control to the market including the common ways that isolates fail along the pipeline. * Indicates the approximate stage in this pipeline that the two most successful isolates from this study have made it to.

Many candidate biocontrol isolates, from a range of bacterial and fungal species, have been tested and shown some impact on crop pathogens at the laboratory scale but very few have been successfully commercialized. This is because there are many ways that a biocontrol candidate can fail along the commercialization pathway, despite showing an ability to suppress a target pathogen *in vitro* (Figure 10). For candidates for development as biocontrol products to be considered successful, a bacterium must: (1) suppress the disease effectively, *in vitro*, *in planta* and under field conditions; (2) survive either within the plant tissues or within the rhizosphere of growing plants while outcompeting native soil microbial communities; (3) be easy to culture and inoculate onto plants to allow for realistic application methods in the field, (4) be produced at a low enough cost to allow growers to apply over large areas while maintaining profitable returns and (5) pass all regulatory and safety requirements (Junaid et al., 2013; Parnell et al., 2016).

Many candidates that show promise *in vitro* fail to sufficiently suppress disease symptoms *in planta* or in field trials. Some promising candidates are difficult to grow at scale on cheap media, resulting in a product that is too expensive to market. Some candidates fail to pass regulatory hurdles to prove that the biocontrol product is safe and has no off-target environmental impacts. Even if all these criteria are met, significant investment is required to market the product and inform growers of its usefulness and place in their integrated pest management strategy.

The best performing isolates in this study have cleared some, but not all these hurdles. Isolates MH71 and MH243 displayed very high levels of suppression of *F. pseudograminearum* both *in vitro and in planta*. They grow readily on simple, cheap media. They can be introduced to the crop via a seed coat. They form spores that are stable at room temperature for long periods. As endophytic species, they have an advantage over other rhizosphere colonizing biocontrol candidates because they take up residence within the plant tissues where they are protected from a range of environmental variables (such as variations in temperature, water content or pH), have ready access to nutrients and water and they do not have to compete with the wider soil microbial community to establish a large enough population to effectively suppress pathogens (Schisler et al., 2002; Franco et al., 2007; Ryan et al., 2008).

However, further work is needed to identify the mechanism of action, to test the effectiveness of the seed-coat treatments for protecting grain yield and quality at the field scale, to establish the safety and toxicology profile of the active compounds and to upscale the production of the spores. Field scale testing, preferably at multiple sites, over multiple seasons in areas that are naturally infested with *Fusarium* pathogens causing crown rot, will be a critical step in verifying the effectiveness of these isolates as biocontrol products. Field trials will not only enable the assessment of the efficacy of disease suppression but will also determine the ability of biocontrol isolates to colonize crop plants and survive under field conditions, which will all contribute to the effectiveness of the biocontrol (Fravel, 2005).

## Conclusion

The screening of 53 isolates from a collection of endophytic Actinobacteria revealed several that are highly inhibitory to the growth of *F. pseudograminearum*. Two isolates in particular, MH71 and MH243, strongly suppressed growth of the fungus on HDPA plates, protected seedlings and mature plants from disease symptoms and led to increases in grain yield compared to disease controls. These two cultures show promise as potential biocontrol candidates because they are also easy to culture, grow on relatively inexpensive media, produce highly durable spores and can be delivered to plants as a seed coat. Further studies are needed to determine optimal growth conditions for scale-up of production, to verify the efficacy of the treatments at the field scale and to determine the mode of action and underlying genetics of biocontrol activity.

## Data Availability Statement

The datasets presented in this study can be found in online repositories. The names of the repository/repositories and accession number(s) can be found below: NCBI GenBank accessions: MZ150495, MZ150496, MZ150497, MZ150498, MZ150499, MZ150500, MZ150501, MZ150502, MZ150503, MZ150504, and MZ150505.

## Author Contributions

MR and CM identified source materials, and isolated, selected and purified cultures of Actinobacteria to produce the endophytic, Actinobacteria collection. CM, CO’S, and LT carried out the experiments. CO’S drafted the manuscript. MR and LT revised and edited the draft. All authors contributed to the conceptualization, experimental design and planning and analysis of the data.

## Conflict of Interest

The authors declare that the research was conducted in the absence of any commercial or financial relationships that could be construed as a potential conflict of interest.
