# Supplementary material for: Developing Actinobacterial Endophytes as Biocontrol Products for Fusarium pseudograminearum in Wheat
Source: Front Bioeng Biotechnol. 2021 Jun 29;9:691770. doi: 10.3389/fbioe.2021.691770 (PMC8276002; doi:10.3389/fbioe.2021.691770)
Supplement: Supplementary file 1 [file Data_Sheet_1.docx]

**Supplementary Material**

Table S1: Counts of viable spores in seed coatings used for the seedling assay and pot trials to assess suppression of F. pseudograminearum by a range of Actinobacteria isolates.

| Actinobacteria isolate | Spore count in seed coating for seedling assay  (viable spores. mL^-1^) | Spore count in seed coating for glasshouse pot trial  (viable spores. mL^-1^) |
| --- | --- | --- |
| 9a | 1.8x10^8^ |  |
| MH20 | 3.5x10^7^ |  |
| MH33 | 4.6x10^7^ |  |
| 36b | 3.08x10^8^ |  |
| MH47 | >300 |  |
| MH51 | 4.9x10^5^ |  |
| MH60 | 1.6x10^7^ |  |
| MH64 | 1.08x10^8^ |  |
| 66b | 3.7x10^7^ | 7.7x10^8^ |
| MH71 | 1.6x10^7^ | 3.3x10^8^ |
| 73a | 9.5x10^6^ |  |
| 73ww | 1.51x10^7^ |  |
| 85b | >3x10^5^ |  |
| MH99 | 1.0x10^5^ |  |
| MH130 | >300 |  |
| MH178 | 7.5x10^7^ |  |
| MH184 | 2.27x10^6^ |  |
| MH187 | 1.62x10^6^ |  |
| MH191 | 2.2x10^7^ |  |
| MH192 | 2.06x10^8^ | 7.6x10^7^ |
| MH243 | 1.0x10^7^ | 4.3x10^8^ |

Table S2: Clearing zones (mm) on half strength PDA plates inoculated, at one end, with one of three *F. pseudograminearum* strains and, at the other end, with an Actinobacteria isolate. nt= not tested. na= not available due to lack of replication.

|  | ***F. pseudograminearum* strain** | | | | | |
| --- | --- | --- | --- | --- | --- | --- |
|  | **CS5642** | | **CS5834** | | **CS3427** | |
| **Actinobacterial isolate identification number** | **Average clearing zone (mm)** | **Std error of mean** | **Average clearing zone (mm)** | **Std error of mean** | **Average clearing zone (mm)** | **Std error of mean** |
| **Control** | 0.0 | 0.0 | 0.0 | 0.0 | 0.0 | 0.0 |
| **9a** | 0.0 | 0.0 | 0.0 | 0.0 | 2.0 | 0.0 |
| **36b** | 0.0 | 0.0 | 4.0 | 0.0 | 2.0 | 0.0 |
| **66b** | 2.5 | 2.0 | 8.3 | 1.7 | 10.5 | 8.6 |
| **73a** | 0.0 | 0.0 | 5.0 | 5.0 | 1.0 | 5.0 |
| **73ww** | 0.0 | 0.0 | 3.3 | 3.3 | 3.0 | 0.0 |
| **85b** | 0.0 | 0.0 | 5.3 | 5.3 | 2.0 | 0.0 |
| **111ww2** | 2.5 | 2.0 | 3.5 | 2.9 | 0.0 | 0.0 |
| **MH12** | 0.0 | 0.0 | nt |  | nt |  |
| **MH20** | 0.0 | 0.0 | nt |  | nt |  |
| **MH30** | 0.0 | 0.0 | nt |  | nt |  |
| **MH33** | 19.3 | 9.7 | 0.0 | 0.0 | 0.0 | 0.0 |
| **MH39** | 10.5 | 8.6 | 0.0 | 0.0 | 0.0 | 0.0 |
| **MH43** | 0.0 | 0.0 | nt |  | nt |  |
| **MH47** | 11.0 | 9.0 | 0.0 | 0.0 | 0.0 | 0.0 |
| **MH50** | 0.0 | 0.0 | nt |  | nt |  |
| **MH51** | 10.0 | 5.3 | 15.0 | na | 13.0 | na |
| **MH56** | 0.0 | 0.0 | nt |  | nt |  |
| **MH57** | 0.0 | 0.0 | nt |  | nt |  |
| **MH60** | 5.0 | 2.1 | 1.5 | 1.2 | 2.0 | 1.6 |
| **MH63** | 29.0 | na | 6.0 | na | 0.0 | 0.0 |
| **MH64** | 29.0 | na | 19.0 | na | 0.0 | 0.0 |
| **MH65** | 0.0 | 0.0 | nt |  | nt |  |
| **MH71** | 25.0 | 2.9 | 20.0 | 5.7 | 29.7 | 10.7 |
| **MH76** | 2.0 | 2.0 | 0.0 | 0.0 | 0.0 | 0.0 |
| **MH85** | 0.0 | 0.0 | nt |  | nt |  |
| **MH94** | 0.0 | 0.0 | nt |  | nt |  |
| **MH99** | 11.0 | 9.0 | 0.0 | 0.0 | 0.0 | 0.0 |
| **MH111** | 0.0 | 0.0 | nt |  | nt |  |
| **MH126** | 0.0 | 0.0 | nt |  | nt |  |
| **MH130** | 0.0 | 0.0 | nt |  | nt |  |
| **MH133** | 4.0 | 2.1 | 6.0 | 0.0 | 2.0 | 0.0 |
| **MH136** | 13.0 | 0.0 | 0.0 | 0.0 | 0.0 | 0.0 |
| **MH137** | 0.0 | 0.0 | nt |  | nt |  |
| **MH145** | 0.0 | 0.0 | nt |  | nt |  |
| **MH164** | 0.0 | 0.0 | nt |  | nt |  |
| **MH176** | 0.0 | 0.0 | nt |  | nt |  |
| **MH178** | 2.0 | 1.5 | 2.0 | 0.0 | nt |  |
| **MH183** | 10.5 | 8.6 | nt |  | nt |  |
| **MH184** | 0.0 | 0.0 | nt |  | nt |  |
| **MH187** | 0.0 | 0.0 | nt |  | nt |  |
| **MH191** | 9.0 | 4.5 | 4.0 | 0.0 | 7.5 | 6.1 |
| **MH192** | 15.0 | 5.0 | 10.7 | 5.5 | 6.0 | 6.0 |
| **MH193** | 0.0 | 0.0 | nt |  | nt |  |
| **MH204** | 0.0 | 0.0 | nt |  | nt |  |
| **MH205** | 0.0 | 0.0 | nt |  | nt |  |
| **MH214** | 0.0 | 0.0 | nt |  | nt |  |
| **MH215** | 0.0 | 0.0 | nt |  | nt |  |
| **MH218** | 0.0 | 0.0 | nt |  | nt |  |
| **MH229** | 0.0 | 0.0 | nt |  | nt |  |
| **MH231** | 0.0 | 0.0 | nt |  | nt |  |
| **MH235** | 0.0 | 0.0 | nt |  | nt |  |
| **MH243** | 29.5 | 8.2 | 29.7 | 7.4 | 7.7 | 5.2 |

Table S3: Genus level identification based on 16s rDNA sequencing, and description of colony morphology of cultures included in phylogenetic analysis (figure 2).

| Isolate Identifier | 16S rDNA genus identification | Colony morphology (mature cultures grown on potato dextrose agar) | BLAST highest identity match(es) | Sequence length | Query coverage (%) | Percent identity (%) | Comment |
| --- | --- | --- | --- | --- | --- | --- | --- |
| 9a | Streptomyces spp | white with grey spores, filiform, umbonate | Streptomyces sp. strain LPA97-4 16S ribosomal RNA gene, partial sequence | 1284 | 100 | 98.62 |  |
| MH60 | Streptomyces spp | white and clear with grey and white spores, irregular, raised | Streptomyces pactum strain AND12 16S ribosomal RNA gene, partial sequence | 1284 | 97 | 98.23 |  |
| MH191 | Streptomyces spp | pink with white and grey spores, filiform, umbonate | Streptomyces sp. strain C1-2 16S ribosomal RNA gene, partial sequence, Streptomyces griseoaurantiacus strain B1 16S ribosomal RNA gene, partial sequence, Streptomyces griseoaurantiacus strain SG-1 16S ribosomal RNA gene, partial sequence, Streptomyces sp. HRTK192 gene for 16S rRNA, partial sequence, Streptomyces griseoaurantiacus strain AC38 16S ribosomal RNA gene, partial sequence | 1284 | 100 | 98.77 | Multiple Blast hits with equal percent identity based on 16s rDNA sequence data. |
| MH192 | Streptomyces spp | pink with white and grey spores, filiform, umbonate | Streptomyces sp. strain C1-2 16S ribosomal RNA gene, partial sequence, Streptomyces griseoaurantiacus strain B1 16S ribosomal RNA gene, partial sequence, Streptomyces griseoaurantiacus strain SG-1 16S ribosomal RNA gene, partial sequence, Streptomyces sp. HRTK192 gene for 16S rRNA, partial sequence, Streptomyces griseoaurantiacus strain AC38 16S ribosomal RNA gene, partial sequence | 1284 | 100 | 98.77 | Multiple Blast hits with equal percent identity based on 16s rDNA sequence data. |
| MH71 | Streptomyces spp | white and grey with grey and black spores, filiform, umbonate | Streptomyces sp. GM-22-10 16S ribosomal RNA gene, partial sequence, Streptomyces sp. E5N344 16S ribosomal RNA gene, partial sequence, Streptomyces sp. MS1 16S ribosomal RNA gene, partial sequence, Streptomyces castelarensis strain BS30 16S ribosomal RNA gene, partial sequence, Select seq CP007153.2 Streptomyces sp. AgN23 chromosome, complete genome | 1284 | 100 | 98.54 | Multiple Blast hits with equal percent identity based on 16s rDNA sequence data. |
| MH243 | Streptomyces spp | white and grey with grey and black spores, filiform, umbonate | Streptomyces sp. GM-22-10 16S ribosomal RNA gene, partial sequence, Streptomyces sp. E5N344 16S ribosomal RNA gene, partial sequence, Streptomyces sp. MS1 16S ribosomal RNA gene, partial sequence, Streptomyces castelarensis strain BS30 16S ribosomal RNA gene, partial sequence, Select seq CP007153.2 Streptomyces sp. AgN23 chromosome, complete genome | 1284 | 100 | 98.54 | Multiple Blast hits with equal percent identity based on 16s rDNA sequence data. |
| MH176 | Microbispora spp | cream with white spores | Microbispora sp. S19 16S ribosomal RNA gene, partial sequence | 1284 | 100 | 98.84 |  |
| MH178 | Microbispora spp | orange / brown with white spores | Microbispora sp. strain CP56 16S ribosomal RNA gene, partial sequence, Microbispora sp. DS32 16S ribosomal RNA gene, partial sequence | 1284 | 100 | 98.92 | Multiple Blast hits with equal percent identity based on 16s rDNA sequence data. |
| MH184 | Microbispora spp | dark orange with white spores | Microbispora sp. strain CP56 16S ribosomal RNA gene, partial sequence, Microbispora sp. DS32 16S ribosomal RNA gene, partial sequence | 1284 | 100 | 99 | Multiple Blast hits with equal percent identity based on 16s rDNA sequence data. |
| MH99 | Micromonospora spp | orange and black, shiny, irregular, entire, raised | Micromonospora sp. strain isolate_SN10_5.1 16S ribosomal RNA gene, partial sequence, Micromonospora sp. strain WMMA1824 16S ribosomal RNA gene, partial sequence, | 1268 | 100 | 99.38 | Multiple Blast hits with equal percent identity based on 16s rDNA sequence data. |
| 66b | Rhodococcus spp | orange, shiny, irregular, entire, raised | Rhodococcus erythropolis CS98 gene for 16S rRNA, partial sequence, Rhodococcus sp. YAZ54 gene for 16S rRNA, partial sequence, Rhodococcus sp. strain H231 16S ribosomal RNA gene, partial sequence, Rhodococcus sp. strain H28 16S ribosomal RNA gene, partial sequence, Rhodococcus erythropolis gene for 16S rRNA, partial sequence, strain: IAM 1414 | 1284 | 100 | 99.3 | Multiple Blast hits with equal percent identity based on 16s rDNA sequence data. |

Figure S1: Temperature and humidity recorded during the glasshouse pot trial.


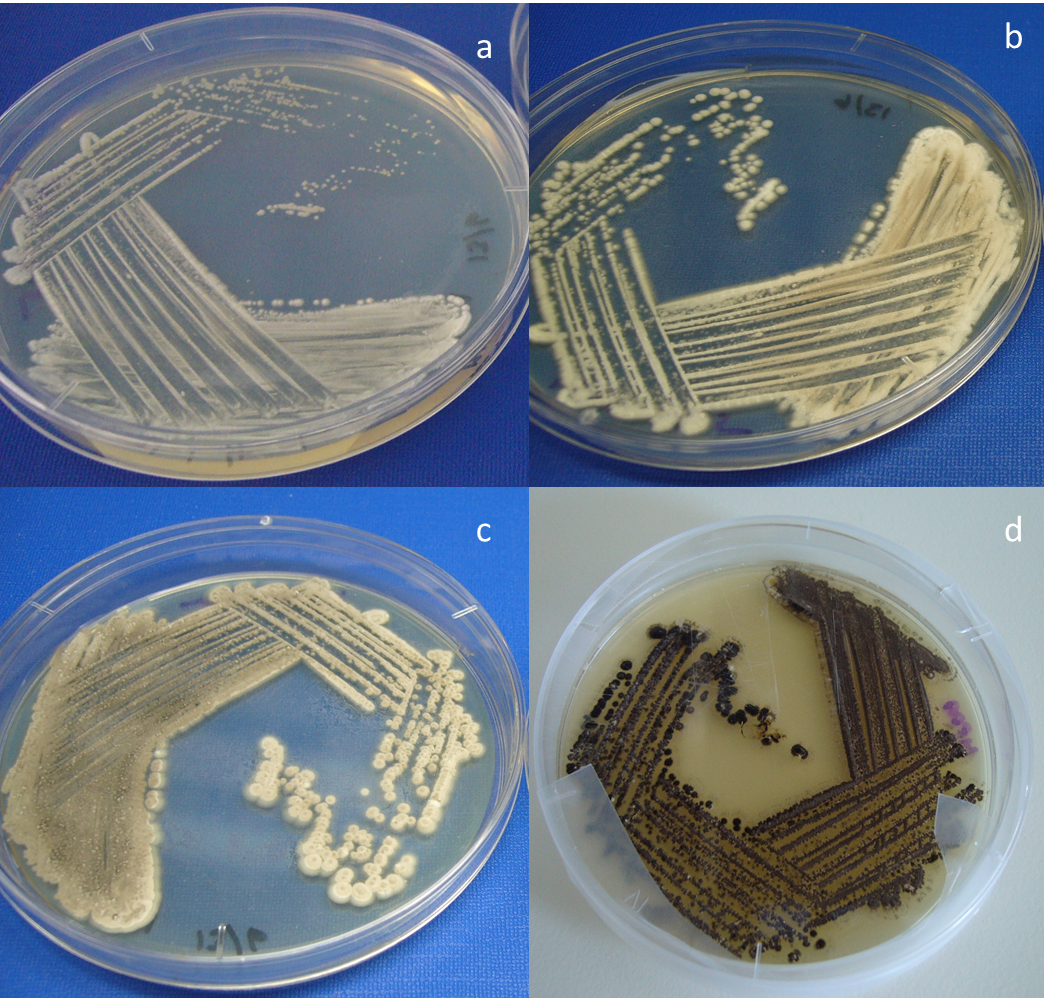


Figure S2: Colony morphology of isolate MH71 at a) 4 days, b) 7 days, c) 14 days and d) 35 days after inoculation


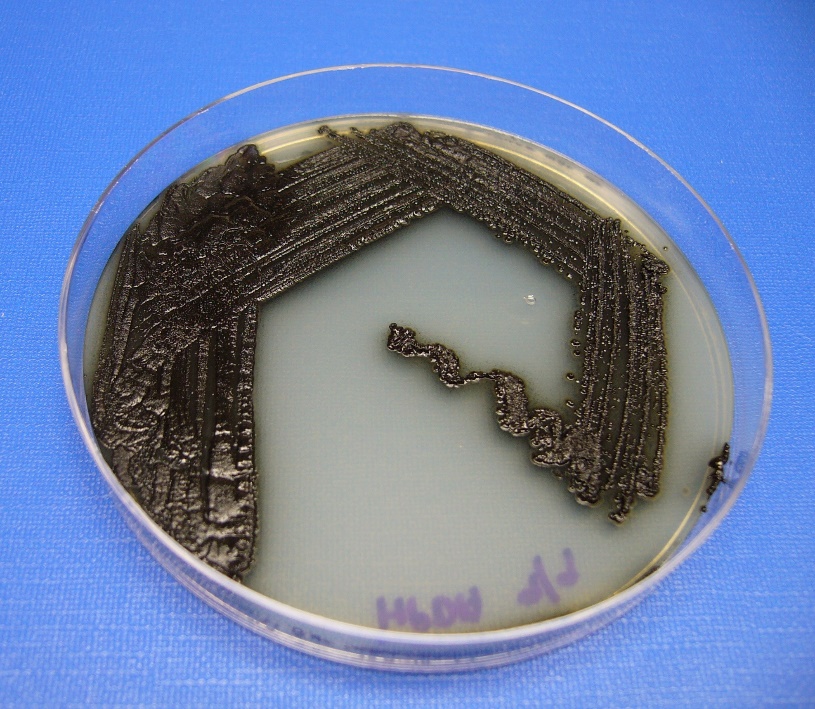

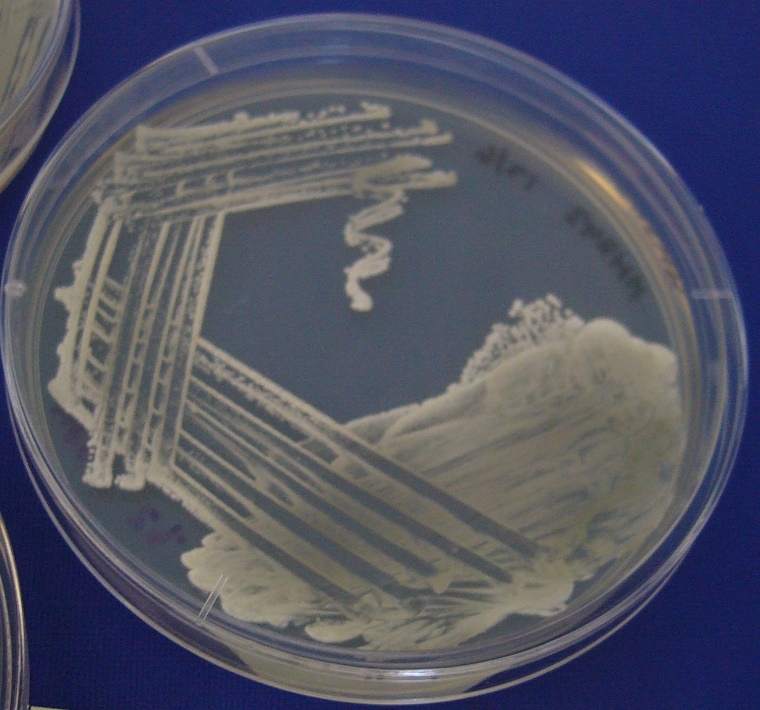

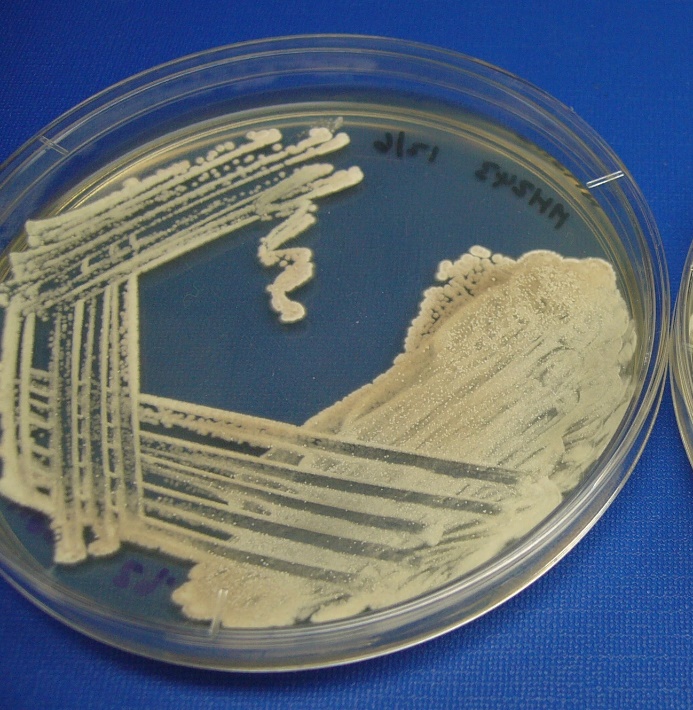

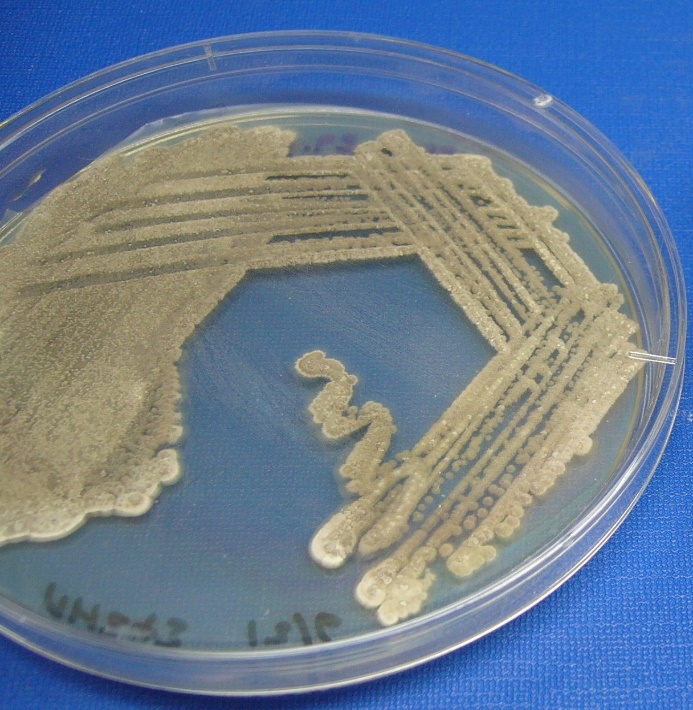


a

b

d

c

Figure S3: Colony morphology of isolate MH243 at a) 4 days, b) 7 days, c) 14 days and d) 35 days after inoculation


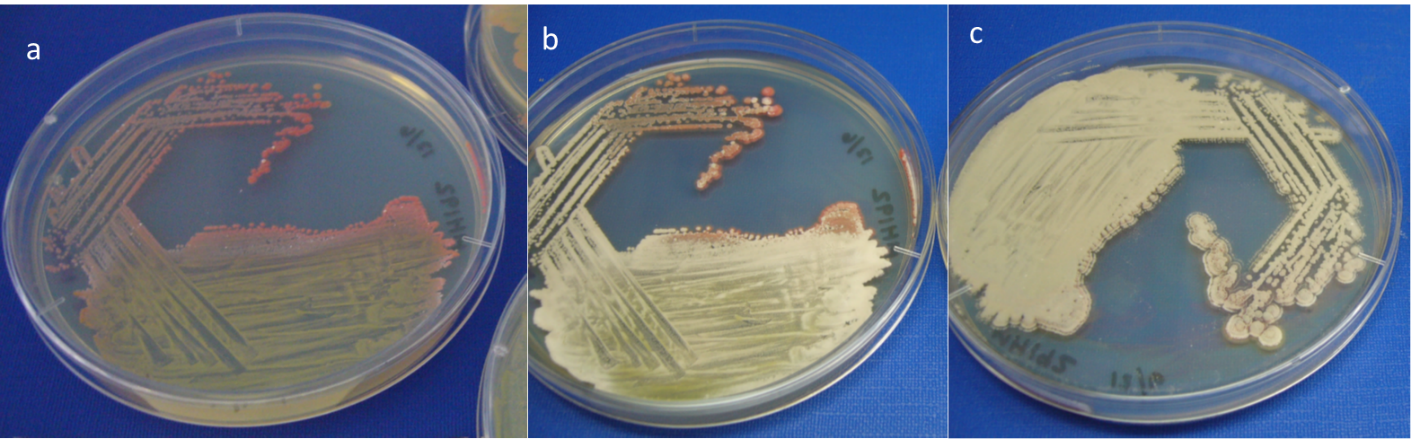


Figure S4: Colony morphology of isolates MH191 and MH192 (very similar visual appearance);

a) 4 days, b) 7days, c) 14 days after inoculation.


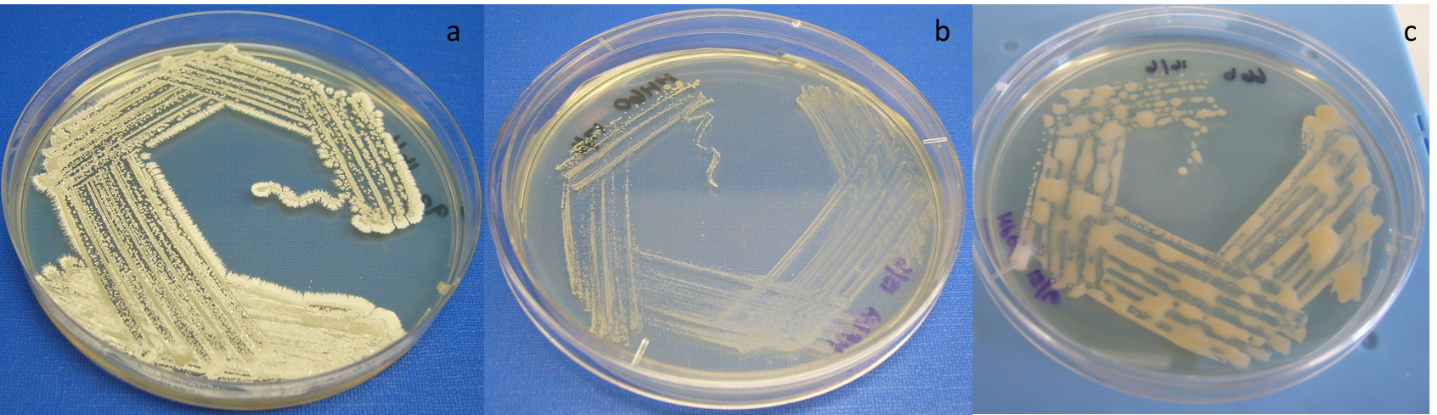


Figure S5: Example colony morphologies of other selected isolates. a) 9a, 8 days after inoculation, b) MH60, 8 days after inoculation, c) 66b, 3 days after inoculation
